# Causal Relationship Between Circulating Inflammatory Cytokines and the Risk of Trigeminal Neuralgia: A Mendelian Randomization Study

**DOI:** 10.1002/brb3.70463

**Published:** 2025-04-07

**Authors:** Hui Shang, Xianqiang Liu, Mengying Bai, Xiao Li, Yuhang Lan, Bingbing Bai, Shuyun Yang, Xianlin Wu, Guocai Li

**Affiliations:** ^1^ Department of Anesthesiology Shenzhen Hospital (Fu Tian) of Guangzhou University of Chinese Medicine Shenzhen People's Republic of China; ^2^ Graduate School Medical School of Chinese PLA Beijing People's Republic of China; ^3^ Reproductive Health Department, Shenzhen Traditional Chinese Medicine Hospital the Fourth Clinical Medical College of Guangzhou University of Chinese Medicine Shenzhen People's Republic of China; ^4^ Department of Anesthesiology The First Affiliated Hospital of Guangzhou University of Chinese Medicine Guangzhou People's Republic of China; ^5^ Cancer Center Shenzhen Hospital (Fu Tian) of Guangzhou University of Chinese Medicine Shenzhen People's Republic of China

**Keywords:** genome‐wide association study, inflammatory cytokine, Mendelian randomization, trigeminal neuralgia

## Abstract

**Background:**

Inflammatory regulators play a fundamental role in the development of trigeminal neuralgia (TN). However, the precise mechanisms and causal relationship with the risk of TN remain poorly understood.

**Methods:**

This study aimed to assess the causal relationship between 41 inflammatory cytokines and TN using Mendelian randomization (MR) analysis. A two‐sample MR approach was utilized, employing genetic variation data on TN from a large publicly available genome‐wide association study (GWAS) comprising 1777 cases of European ancestry and 360,538 controls. Additionally, summary data from a GWAS on inflammatory cytokines, comprising 8293 healthy participants, were utilized. The causal relationship between exposure and outcome was primarily assessed using the inverse variance weighted (IVW) method, accompanied by sensitivity analyses.

**Results:**

The study revealed an association between increased risk of TN and cutaneous T cell‐attracting chemokine（CTACK） (odds ratio [OR] = 1.187; 95% confidence interval [CI], 1.041–1.35; *p* = 0.01) and interferon (IFN)‐gamma（MIG） (OR = 1.232; 95% CI, 1.080–1.449; *p* = 0.01), while interleukin (IL)‐16 (OR = 0.823; 95% CI, 0.685–0.989; *p* = 0.03) and interferon (IFN)‐G (OR = 0.779; 95% CI, 0.612–0.992; *p* = 0.04) were associated with decreased risk of TN. Notably, no potential effect of TN on inflammatory factors was observed.

**Conclusion:**

This study provides novel insights into the pathogenesis of TN, highlighting the crucial role of inflammatory cytokines in TN risk.

**Significance:**

This study advances our understanding of TN by using MR to identify the causal roles of specific inflammatory cytokines. These results underscore the importance of inflammation in TN development and suggest potential targets for new treatments.

## Graphical abstract

This Mendelian randomization study identifies CTACK and MIG as risk‐increasing cytokines, and interleukin (IL)‐16 and interferon (IFN)‐G as protective factors for trigeminal neuralgia. Findings highlight inflammation's causal role in trigeminal neuralgia (TN) pathogenesis and suggest novel therapeutic targets, advancing mechanistic understanding of this debilitating condition.

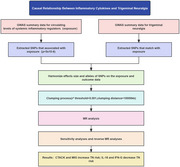

## 1 Introduction

Trigeminal neuralgia (TN) is a chronic neuropathic pain condition characterized by abnormal pain, nociceptive hypersensitivity, and spontaneous pain (Lambru et al. 2021). Its underlying cause is often a lesion or dysfunction of the trigeminal nerve, responsible for sensation in the face (Khawaja and Scrivani 2023). Despite numerous therapeutic modalities, clinical outcomes have remained suboptimal. The trigeminal caudate nucleus (TNC), the first central relay station for trigeminal nerve signals, plays a crucial role in processing pain signals from the face (Bertels et al. 2022). Several studies have shown that primary cultures of astrocytes with tumor necrosis factor (TNF)‐α induce significant increases in several chemokines, such as CCL2 and CXCL1, which may contribute to central sensitization and chronic pain (Sharma et al. 2024; Yang et al. 2023). Monocytes migrate to inflamed or damaged tissues, creating a long‐term inflammatory microenvironment in chronic inflammatory diseases. This migration is facilitated by chemokine receptors like CCR2, CXCR5, CCR5, and CCR1, which respond to CCL2 and CCL3 gradients. These monocytes secrete various inflammatory cytokines and chemokines, including CCL2, interleukin (IL)‐8, IL‐6, IL‐1β, and TNF‐α (Batbold et al. 2017). Targeted inhibition of signaling molecules expressing glial cells, such as TNF‐α, IL‐1β, and CCL2, has been shown to reduce neuropathic pain (Sharma et al. 2024; Yu et al. 2022).

The pathogenesis of TN is thought to involve a pivotal role for inflammatory cytokines (Liu et al. 2019). They act as immune system messengers, orchestrating, and modulating responses that influence the onset, progression, and resolution of inflammation. Although epidemiological studies have explored the association between inflammatory cytokines and TN, results remain inconsistent. Some studies suggest a potential link between TN risk and altered levels of IL‐1β, IL‐6, IL‐8, and TNF‐α (Marek et al. 2019; Ostertag et al. 2023; Patil and Testarelli 2021). While others revealed no associations (Lu et al. 2021). Furthermore, the observed associations between inflammatory cytokines and TN risk are susceptible to confounding by various factors, including limited follow‐up durations, small sample sizes, and potential reverse causality (Smith and Ebrahim 2003). These limitations may lead to spurious conclusions. Consequently, establishing a causal relationship between inflammatory cytokines and TN risk remains a challenging task, necessitating more rigorous and well‐controlled studies to elucidate the underlying mechanisms.

Mendelian randomization (MR) analysis offers a distinctive and valuable approach to infer causality by leveraging genetic variation (Sekula et al. 2016). The random allocation of alleles during meiosis enables MR to mitigate the influence of traditional confounding variables, thereby generating robust evidence for causal inference (Burgess et al. 2015). Moreover, utilizing bidirectional MR techniques allows researchers to comprehensively evaluate the associations between instruments and exposures and instruments and outcomes across different population samples. This approach significantly enhances the practicality and efficacy of the tests (Hartwig et al. 2016).

In light of aforementioned evidence, this study aimed to conduct MR analyses by identifying representative instrumental variables (IVs) to elucidate the causal association between inflammatory cytokines with TN. Through this work, we aimed to provide more epidemiological evidence for the relevant fields and contribute to the development of novel treatment approaches that can improve patient outcomes.

## 2 Methods and Materials

### 2.1 MR Assumptions

**FIGURE 1:**
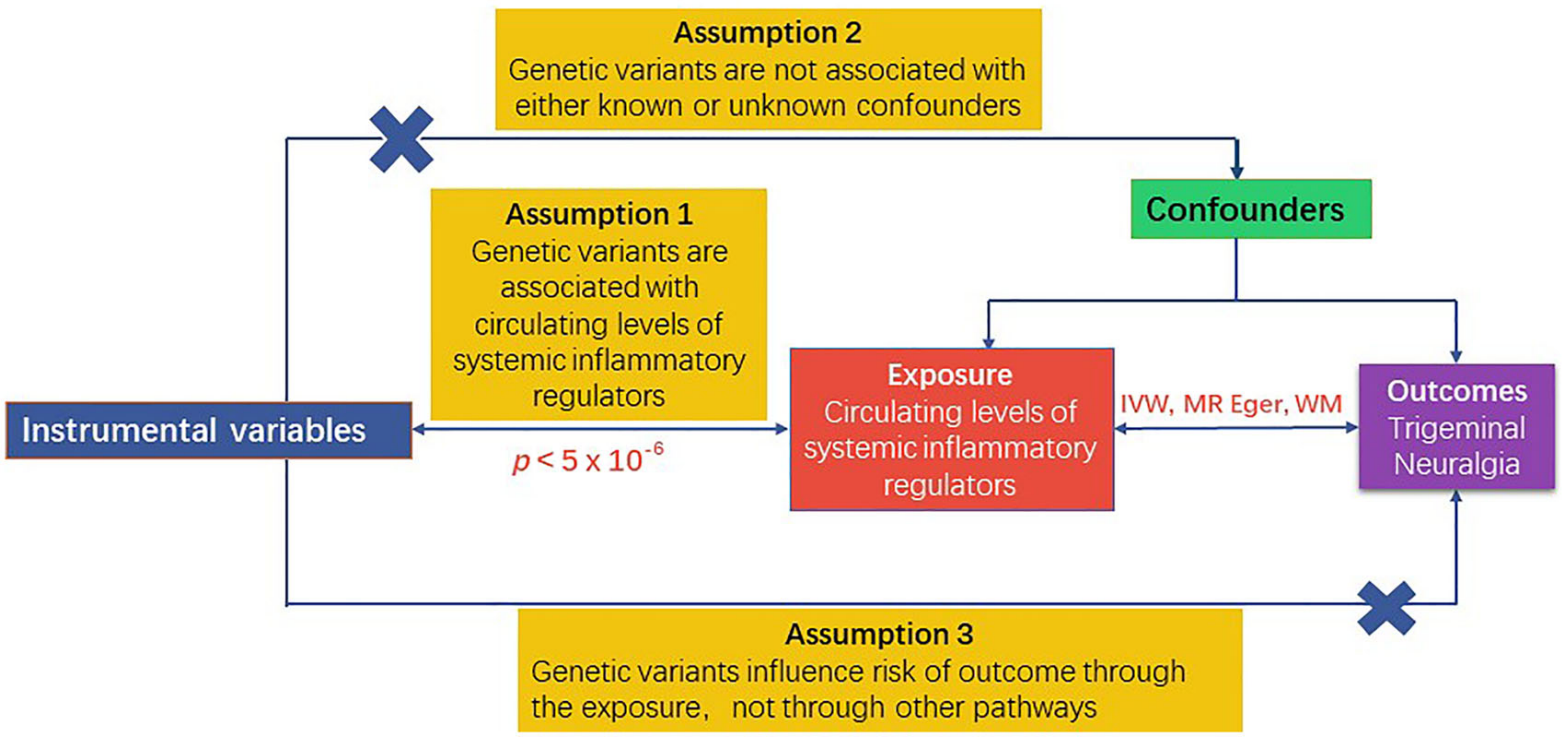
The directed acyclic graph (DAG) representing the Mendelian randomization (MR) framework employed to investigate the causal relationship between the circulating levels of systemic inflammatory regulators and trigeminal neuralgia.

The MR analysis is predicted on three foundational assumptions (Figure 1), namely, relevance, independence, and exclusion restrictions (Bowden and Holmes 2019). The first assumption, relevance, requires that the selected genetic variants exhibit a significant association with the exposure, thereby establishing a credible link between the two. The second assumption, independence, posits that these genetic variants are unconfounded by any factors that might influence the relationship between the exposure and the outcome, thereby ensuring that the association is not biased by extraneous variables. The third assumption, exclusion restrictions, asserts that the genetic variants exert their effect on the outcome solely through the exposure, thereby precluding the possibility of pleiotropic effects. LDlinkR R package will be used to search the phenotype of each single nucleotide polymorphism (SNP) to exclude the effect of confounders, which were not found to be associated with confounders in the IVs extracted for us (Supporting Information Table 1).

### 2.2 Data Source

Data used for MR analysis were extracted from two publicly accessible summary‐level genome‐wide association study (GWAS). This study was primarily conducted in accordance with the Strengthening the Reporting of Observational Studies in Epidemiology Using Mendelian Randomization guideline. The ethical committees of each participating institution approved the study, and written informed consent was obtained from all participants. Consequently, the study complied with all requisite ethical guidelines, and no additional ethical approvals were deemed necessary.

Data on exposure were extracted from a meta‐analysis of 41 inflammatory cytokines‐associated GWAS data across three independent Finnish cohorts, namely, the Cardiovascular Risk in Young Finns study (YFS) and the 1997 and 2002 FINRISK studies. Data on outcome (TN) were derived from FinnGen database This study included 3690 TN cases and 361,055 controls of European ancestry. The TN cases were diagnosed based on ICD9 and ICD10 criteria.

The two datasets utilized in the MR analysis were sourTN from publicly accessible summary GWAS data. The cases for TN were obtained from a database in Finland, which included 3690 cases and 361,055 European ancestry controls. Patients with TN were diagnosed based on the ICD9 and ICD10 criteria. Through an extensive meta‐analysis of cytokine‐associated GWAS across three independent cohorts, genetic predictors for 41 systemic inflammation regulators were derived. These cohorts included a total of 8293 Finnish participants from the Cardiovascular Risk in YFS and the “FINRISK” studies (FINRISK1997 and FINRISK2002). Furthermore, cytokines were normalized through a two‐step inverse transformation. Additionally, to assess individual relationships between 10.7 million genetic polymorphisms and the concentrations of these cytokines, an additive genetic model This model was adjusted for covariates including age, sex, body mass index (BMI), and the first 10 genetic principal components, ensuring the control of potential confounding factors (Bowden and Holmes 2019). The average age of participants in the YFS was 37 years, while that in the FINRISK survey was 60 years. The individuals selected for the exposure group and the outcome group were mutually exclusive, with no overlapping population samples.

### 2.3 Instrumental Variable Selection

For the selection of IVs, a genome‐wide significance threshold of *p* < 5 × 10^−8^ was used. In cases where cytokines‐associated SNPs were limited, we applied a more lenient threshold of *p* < 5 × 10^−6^ to ensure an adequate number of IVs. We addressed linkage disequilibrium among SNPs by employing a clumping procedure with window size of 10,000 kb and an *r* ^2^ threshold of 0.001 based on the European 1000 Genome reference panel. Palindromic SNPs were excluded to prevent alignment ambiguities. The strength of IVs was assessed by the *F*‐statistics, where *F* < 10 indicated weak strength of IVs (Bowden and Holmes 2019).

### 2.4 Statistical Analysis

Heterogeneity and pleiotropy among IVs were evaluated using Cochrane's *Q*‐test and MR‐Egger intercept test, respectively. MR‐Egger regression and MR‐PRESSO analyses were additionally conducted to further examine pleiotropy. We mainly used inverse‐variance weighted method to performed MR analysis. If heterogeneity was detected, the multiplicative random‐effect was used, otherwise, the fixed‐effect model was adopted. When only one IV were identified for MR analysis, Wald ratio method was applied (Georgakis et al. 2019; Perry et al. 2021). Leave‐one‐out analysis was employed to assess the influence of individual SNPs on the overall causal association. To mitigate the risk of false‐positive findings, a Bonferroni‐corrected significance threshold of *p* < 0.0012 (0.05/41) was adopted. Results with *p*‐values between 0.0012 and 0.05 were considered indicative of potential associations (Georgakis et al. 2019).

We further investigated the reverse causal relationship, with TN as the exposure and inflammatory cytokines as outcomes, using the similar IV selection and analytical procedures

**FIGURE 2:**
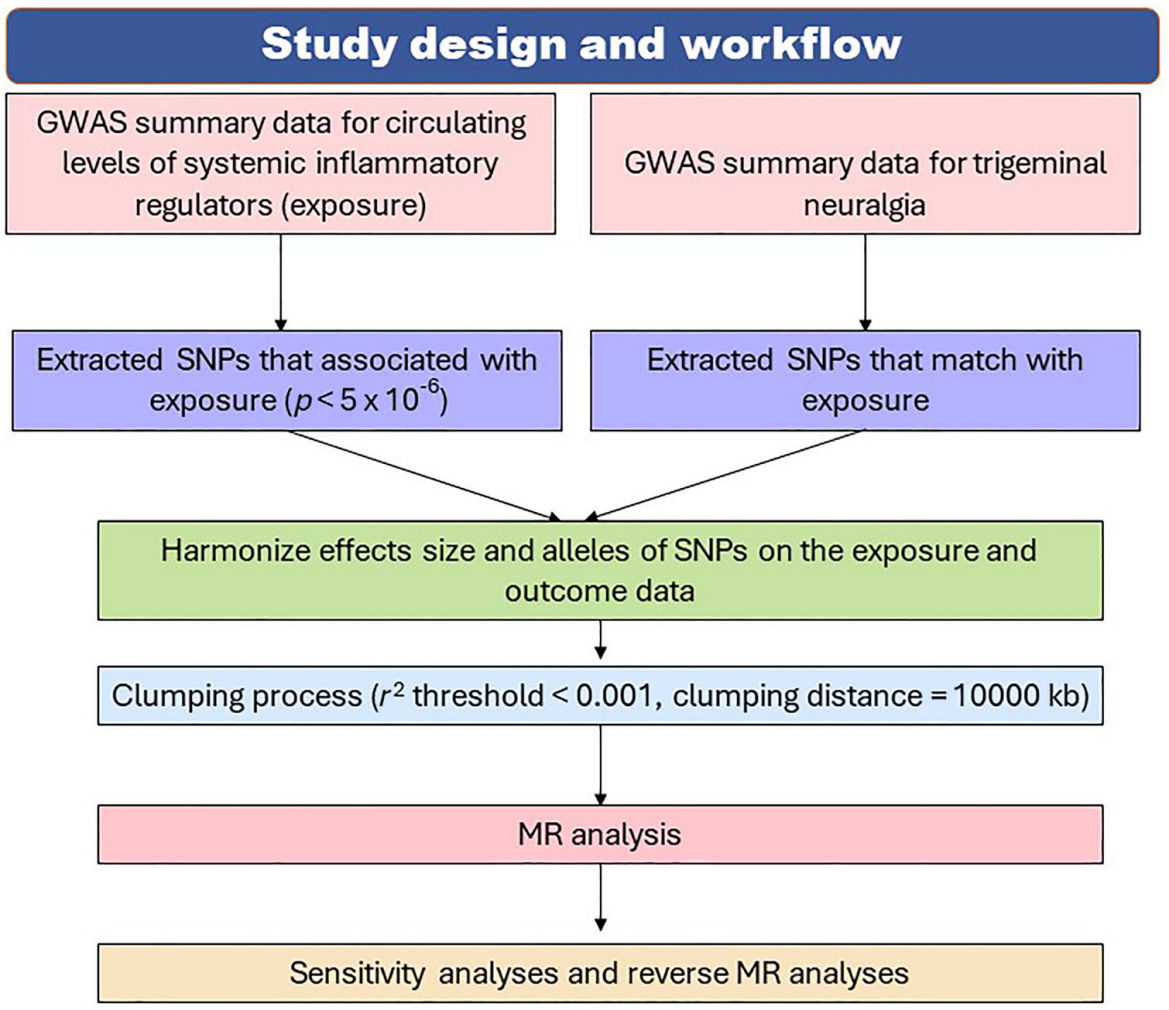
Flow chart of this study. GWAS, genome‐wide association study; MR, Mendelian randomization; SNPs, single nucleotide polymorphisms.

All analyses were conducted in R software using the TwoSampleMR (v 0.5.6) and MR‐PRESSO (Verbanck et al. 2018) packages. All reported *p*‐values are two‐tailed. The analysis flowchart is shown in Figure 2.

## 3 Results

### 3.1 The Causal Effect of Inflammatory Cytokines on TN Risk

For all 41 inflammatory modulators, the causal effect of systemic inflammatory regulation on TN risk reached statistical significance at a less stringent threshold (*p* < 5 × 10^−6^). The *F*‐statistics for these modulators ranged from 20.7 to 782.2, with all *F*‐test values exceeding 10, indicating the absence of significant weak instrument bias (Supporting Information Table 2).

**FIGURE 3:**
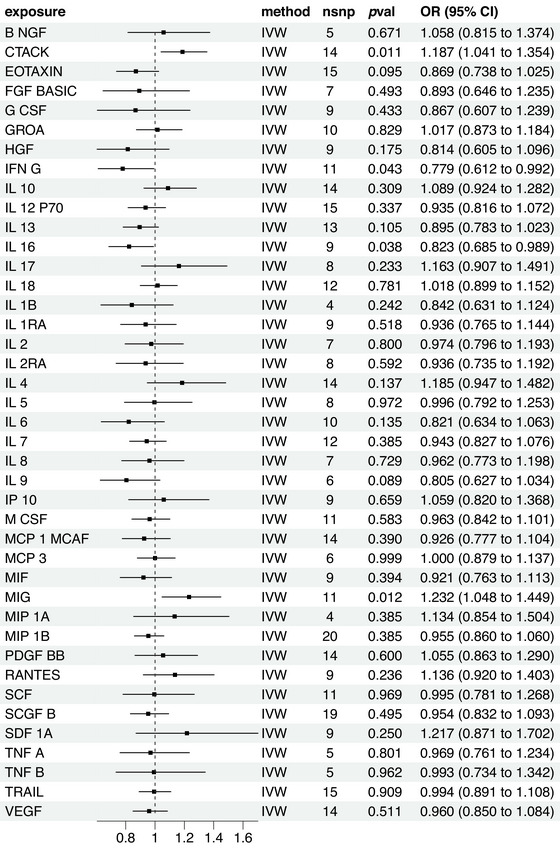
Causal correlations of 41 inflammatory cytokines on trigeminal neuralgia. The change in the odds ratio (OR) of celiac disease per one‐standard deviation (SD) rise in the cytokine level is shown by OR and 95% confidence interval (CI). *p*‐value 0.05/41 = 0.0012 was found significant after multiple‐comparison correction. The results from inverse variance weighted method were shown for all cytokines.

**FIGURE 4:**
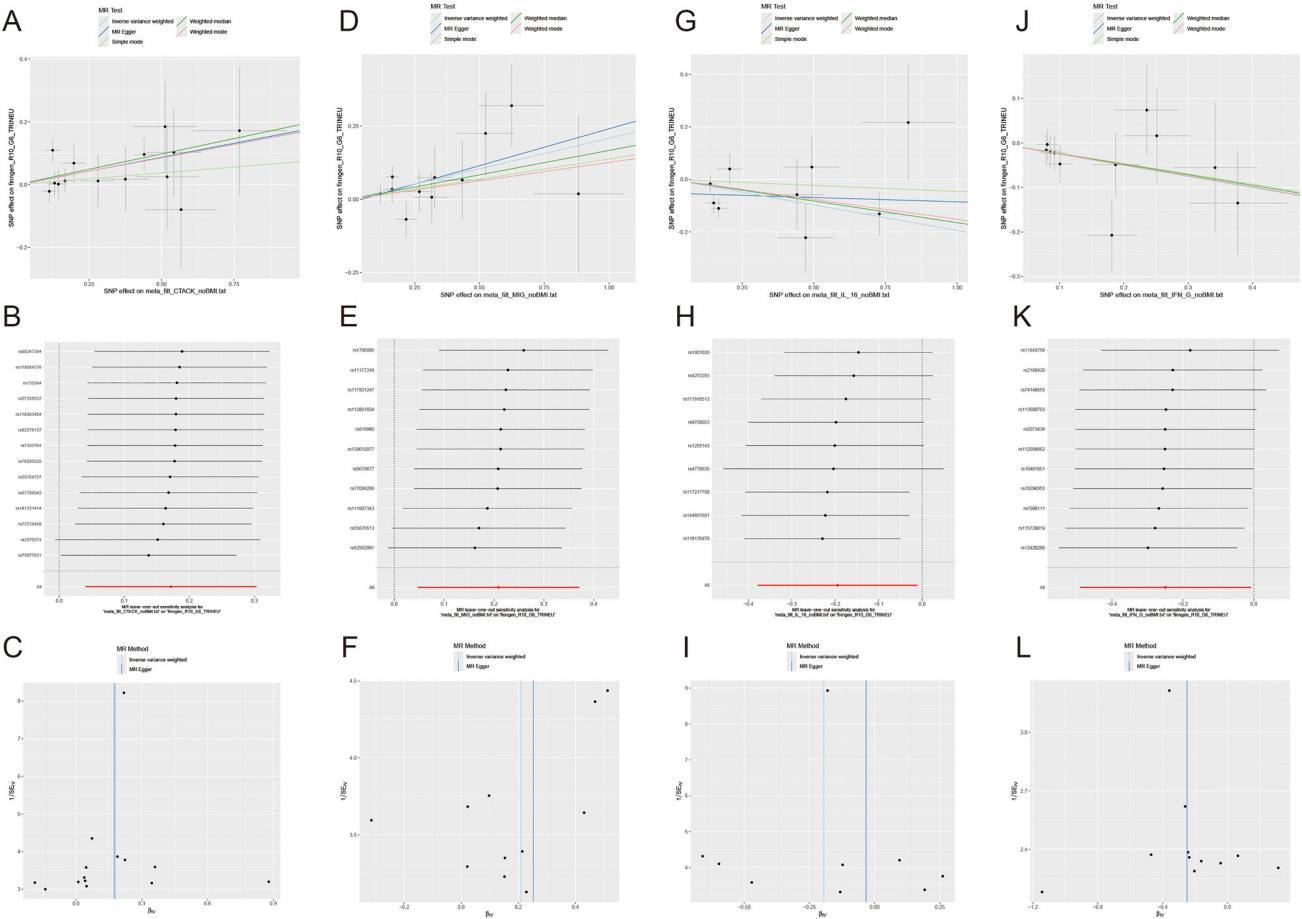
Scatter plots and funnel plots of Mendelian randomization (MR) analyses for CTACK (A, B), MIG (D, E), interleukin (IL)‐16 (G, H), and interferon (IFN)‐G (J, K) in trigeminal neuralgia. The funnel plots show the inverse variance weighted MR estimate of each cytokine single‐nucleotide polymorphism with trigeminal neuralgia versus 1/standard error (1/SEIV; C, F, I, L).

Four inflammatory factors, including CTACK, MIG, IL‐16, and interferon (IFN)‐G exhibited a causal relationship with TN. As depicted in Figure 3, A notable association was noted between the TN risk and CTACK and significant effects were observed in the inverse variance weighted (IVW) method (odds ratio [OR] = 1.187; 95% confidence interval [CI], 1.041–1.35; *p* = 0.01) in the weighted median method (OR = 1.216; 95% CI, 1.014–1.458; *p* = 0.034). In addition, the MR‐Egger method (OR = 1.194; 95% CI, 0.935–1.524; *p* = 0.179) did not detect a significant effect, but the direction of the effect was congruent with IVW. MIG was found to render a significant impact (OR = 1.232; 95% CI, 1.080–1.449; *p* = 0.01). While the MR‐Egger analysis (OR = 1.287; 95% CI, 0.924–1.793; *p* = 0.169) and the weighted median method (OR = 1.182; 95% CI, 0.954–1.464; *p* = 0.126) did not reveal a significant impact. However, all methods showed consistency in the direction of the effect, as observed in IVW analysis. IL‐16 demonstrated a significant negative correlation with TN risk (OR = 0.823; 95% CI, 0.685–0.989; *p* = 0.03), while the MR‐Egger analysis (OR = 0.848; 95% CI, 0.699–1.028; *p* = 0.094) and the weighted median method (OR = 0.968; 95% CI, 0.718–1.304; *p* = 0.835) indicated a similar effect, did not reveal a significant impact. However, all methods showed consistency in the direction of the effect, as observed in IVW analysis. IFN‐G was inversely associated with TN risk (OR = 0.779; 95% CI, 0.612–0.992; *p* = 0.04), with the weighted median method (OR = 0.788; 95% CI, 0.576–1.079; *p* = 0.137) and MR‐Egger analysis (OR = 0.779; 95% CI, 0.470–1.290; *p* = 0.358) not indicating significant effects. Nevertheless, their impact direction aligned with that observed in the IVW analysis (Figure 4A,C,F,G,).

The robustness assessment revealed no significant pleiotropy in the MR‐Egger intercept, and MR‐PRESSO did not identify any outliers (Supporting Information Table 3). To provide a more intuitive representation of the aforementioned relationships, scatterplots and funnel plots of the MR analysis of CTACK, MIG, IL‐16, and IFN‐G with TN risk are presented in Figure 4.

**FIGURE 5:**
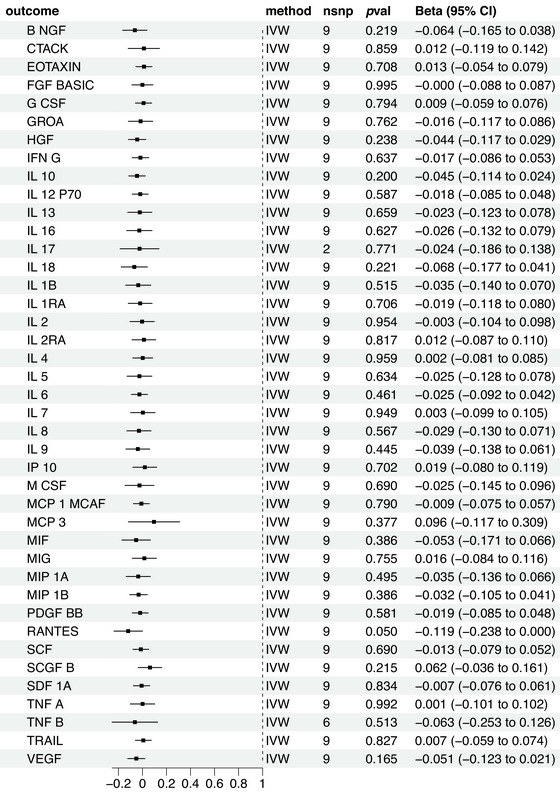
Causal correlations of trigeminal neuralgia on 41 inflammatory cytokines. The change in the standard deviation (SD) of inflammatory cytokines per log odds increase in trigeminal neuralgia is represented by beta and the 95% confidence interval (CI). The results from inverse variance weighted method were shown for all cytokines.

To evaluate reverse causal effects, this study identified 34 SNPs with strong and independent associations with TN at a threshold of *p* < 5 × 10^−6^. As SNP data for certain cytokines could not be obtained through proxy SNPs and harmonization, different quantities of SNPs were employed for the analysis of various cytokines. Using the IVW approach, we did not observe a significant association between any inflammatory factor and TN (Figure 5).

The results showed good performance in the robustness assessment. The MR‐Egger intercept did not exhibit significant pleiotropy, and MR‐PRESSO did not identify any outliers (Supporting Information Table 4).

## 4 Discussion

In this study, we employed MR at the summary genetic level to investigate the causal relationship between systemic inflammatory regulators and the risk of TN. Our findings suggest that elevated levels of CTACK and MIG increase the risk of TN, while higher levels of IL‐16 and IFN‐G confer a protective effect. These results underscore the importance of inflammatory pathways in TN pathogenesis and highlight potential targets for therapeutic intervention.

Inflammation has been recognized as a pivotal mechanism in modulating neuropathic pain, presenting a potential therapeutic target (Panigrahy et al. 2021). Overexpression of chemokines can lead to chronic inflammation and contribute to neuropathic pain by exerting effects on neural tissue (Pawlik and Mika 2023). The observed associations between CTACK, MIG, and TN risk align with previous studies that have implicated these cytokines in pain modulation and neuroinflammation (Liu et al. 2019; Ostertag et al. 2023). CTACK, also known as CCL27, is primarily involved in T‐cell trafficking to inflamed tissues, and its elevated levels may exacerbate peripheral and central sensitization mechanisms underlying TN (Patil and Testarelli 2021). A recent study identified key genes and pathways associated with pain responses in sheep and goats using RNA‐sequencing analysis. The study revealed that N‐methyl‐D‐aspartate (NMDA) receptor signaling, inflammatory responses, immune responses, and *CCL27*, *GluA2*, and *SCN3A* were crucial genes that potentially regulate chronic pain processes in sheep (Deng et al. 2018). These candidate genes could serve as potential targets for developing novel treatments for chronic pain.

Similarly, MIG (CXCL9) is a chemokine induced by IFN‐G that plays a critical role in leukocyte recruitment and may contribute to neuroinflammatory processes in TN (Yang et al. 2023). Studies have found that activation of the spinal cord CXCL0/CXCR3 pathway mediates bone cancer pain (BCP), and inhibition of spinal cord CXCR3, as well as downstream signaling molecules such as Akt and ERK1/2, can alleviate mechanical allodynia (Guo and Gao 2015). Reports have shown that IncRNA *NONRATT021203.2* upregulation in thedorsal root ganglion（DRG） is associated with bone cancer‐induced pain (Sun et al. 2020). Research has further revealed that the G protein‐coupled receptor GPR151 mediates Gβγ/extracellular signal‐regulated kinase (ERK) pathway activation in the DRG, contributing to the pathogenesis of TN. *GPR151* mutations result in reduced expression of numerous proinflammatory genes, including *CCL5*, *CCL7*, *CXCL9*, and *CXCL10*, in wild‐type mice (Jiang et al. 2021). Therefore, MIG represents a potential target for the treatment of TN.

In contrast, IL‐16 and IFN‐G appear to exert protective effects against TN. IL‐16, a multifunctional cytokine, modulates immune responses by acting as a chemoattractant for CD4+ T cells and modulating the release of other proinflammatory cytokines (Sharma et al. 2024). Although IL‐16 is a recognized proinflammatory cytokine, studies have reported its role as an anti‐inflammatory cell signaling molecular in rheumatoid arthritis (Klimiuk et al. 1999; Yoon et al. 2020). The inverse association between IL‐16 and TN risk suggests a potential anti‐inflammatory role that warrants further investigation.

IFN‐G, a key cytokine in the Th1 immune response, has been shown to have complex effects on neuroinflammation and pain perception, potentially reducing TN risk through anti‐inflammatory and immunomodulatory pathways (Batbold et al. 2017). IFNs are cell signaling proteins that play important roles in immunity, endocrinology, and neurology, including the peripheral and central nervous systems (Tanemura et al. 2022). The activation of astrocytes triggered by IFN‐γ is associated with the manifestation of trigeminal neuropathic pain (Asano et al. 2023).

Our study has several strengths, including the use of large, well‐characterized GWAS datasets and robust MR methods to infer causality. The bidirectional nature of our analysis further strengthens the evidence for a causal relationship between inflammatory cytokines and TN risk. However, some limitations should be noted. First, the generalizability of our findings may be restricted due to all data were from European populations, which may not be applicable to represent other populations. Future research should include other populations to validate our findings. Second, we only explored the causal association between inflammation cytokines and TN, while gene–environment interactions may provide further insight into related field, which is needed further investigation. Finally, despite employing methods such as MR‐Egger and MR‐PRESSO to evaluate bias, some residual confounding cannot fully avoid. We recommend that future studies refine their control strategies and integrate clinical data to validate our findings more thoroughly.

## 5 Conclusion

In conclusion, this MR study provides novel insights into the role of inflammatory cytokines in TN pathogenesis, identifying CTACK, MIG, IL‐16, and IFN‐G as key players. Importantly, no evidence was observed supporting an impact of TN on the levels of inflammatory factors. These findings open new avenues for research into targeted therapeutic strategies that modulate specific inflammatory pathways to improve TN management and patient outcomes.

## Author Contributions

Hui Shang and Guocai Li conceived and supervised the study and revised the paper. Hui Shang and Xianqiang Liu formatted the paper and wrote different parts of the paper. Mengying Bai and Hui Shang organized the figures. All the authors have read and approved the article.

## Ethics Statement

Ethical review and approval were waived for this study because no individual patients were directly involved in the overall process of our study. Our study was based only on publicly available GWAS data.

## Conflicts of Interest

The authors declare no conflicts of interest.

### Peer Review

The peer review history for this article is available at https://publons.com/publon/10.1002/brb3.70463.

## Supporting information



Supporting Information.

Supporting Information.

Supporting Information.

Supporting Information.

## Data Availability

All data involved in the current study are publicly available data from individual reference papers.
