# Supplementary material for: Causal Relationship Between Circulating Inflammatory Cytokines and the Risk of Trigeminal Neuralgia: A Mendelian Randomization Study
Source: Brain Behav. 2025 Apr 7;15(4):e70463. doi: 10.1002/brb3.70463 (PMC11975542; doi:10.1002/brb3.70463)
Supplement: Supplementary file 3 — Supporting Information. [file BRB3-15-e70463-s004.docx]

**Supplementary Table 3. Heterogeneity and horizontal pleiotropy tests of forty-one inflammation cytokines on trigeminal neuralgia.**

| **Exprosure** | **Q1 pval** | **Q2 pval** | **intercept** | **Intercept pval** | **MR-PRESSO**  **Global** |
| --- | --- | --- | --- | --- | --- |
| B_NGF | 0.239 | 0.337 | -0.051 | 0.661 | 0.374 |
| CTACK | 0.666 | 0.740 | -0.002 | 0.957 | 0.776 |
| EOTAXIN | 0.872 | 0.911 | 0.002 | 0.941 | 0.915 |
| FGF_BASIC | 0.223 | 0.300 | -0.027 | 0.677 | 0.310 |
| G_CSF | 0.029 | 0.048 | 0.005 | 0.920 | 0.079 |
| GROA | 0.025 | 0.038 | -0.024 | 0.720 | 0.084 |
| HGF | 0.233 | 0.294 | 0.022 | 0.642 | 0.333 |
| IFN_G | 0.665 | 0.750 | 0.000 | 0.999 | 0.766 |
| IL_10 | 0.707 | 0.647 | 0.029 | 0.228 | 0.584 |
| IL_12_P70 | 0.963 | 0.978 | 0.002 | 0.921 | 0.977 |
| IL_13 | 0.116 | 0.115 | -0.033 | 0.375 | 0.158 |
| IL_16 | 0.129 | 0.082 | -0.052 | 0.230 | 0.105 |
| IL_17 | 0.744 | 0.505 | 0.062 | 0.145 | 0.526 |
| IL_18 | 0.996 | 0.991 | 0.031 | 0.366 | 0.993 |
| IL_1B | 0.338 | 0.518 | -0.036 | 0.789 | 0.543 |
| IL_1RA | 0.329 | 0.316 | 0.047 | 0.328 | 0.313 |
| IL_2 | 0.805 | 0.335 | 0.069 | 0.086 | 0.248 |
| IL_2RA | 0.009 | 0.014 | 0.014 | 0.753 | 0.048 |
| IL_4 | 0.976 | 0.973 | -0.025 | 0.415 | 0.971 |
| IL_5 | 0.105 | 0.141 | -0.029 | 0.627 | 0.147 |
| IL_6 | 0.645 | 0.590 | 0.039 | 0.265 | 0.627 |
| IL_7 | 0.297 | 0.363 | 0.015 | 0.697 | 0.399 |
| IL_8 | 0.623 | 0.743 | -0.002 | 0.957 | 0.739 |
| IL_9 | 0.728 | 0.826 | -0.025 | 0.742 | 0.825 |
| IP_10 | 0.191 | 0.041 | -0.091 | 0.076 | 0.053 |
| M_CSF | 0.993 | 0.997 | 0.001 | 0.975 | 0.998 |
| MCP_1_MCAF | 0.367 | 0.435 | 0.013 | 0.728 | 0.389 |
| MCP_3 | 0.976 | 0.722 | -0.068 | 0.197 | 0.696 |
| MIF | 0.851 | 0.504 | -0.076 | 0.087 | 0.501 |
| MIG | 0.503 | 0.589 | -0.011 | 0.775 | 0.574 |
| MIP_1A | 0.346 | 0.464 | -0.047 | 0.585 | 0.522 |
| MIP_1B | 0.112 | 0.141 | 0.007 | 0.768 | 0.207 |
| PDGF_BB | 0.061 | 0.081 | 0.013 | 0.679 | 0.098 |
| RANTES | 0.452 | 0.226 | 0.086 | 0.092 | 0.260 |
| SCF | 0.338 | 0.419 | 0.011 | 0.787 | 0.430 |
| SCGF_B | 0.097 | 0.084 | 0.034 | 0.281 | 0.087 |
| SDF_1A | 0.127 | 0.184 | -0.007 | 0.870 | 0.190 |
| TNF_A | 0.318 | 0.464 | -0.013 | 0.825 | 0.512 |
| TNF_B | 0.006 | 0.002 | -0.082 | 0.370 | 0.006 |
| TRAIL | 0.343 | 0.308 | -0.028 | 0.246 | 0.343 |
| VEGF | 0.195 | 0.250 | 0.004 | 0.848 | 0.359 |

Q1 pval: p value of Q test from IVW method; Q2 pval: p value of Q test from MR-Egger method
