# Supplementary material for: Causal Relationship Between Circulating Inflammatory Cytokines and the Risk of Trigeminal Neuralgia: A Mendelian Randomization Study
Source: Brain Behav. 2025 Apr 7;15(4):e70463. doi: 10.1002/brb3.70463 (PMC11975542; doi:10.1002/brb3.70463)
Supplement: Supplementary file 4 — Supporting Information. [file BRB3-15-e70463-s001.docx]

**Supplementary Table 4. Heterogeneity and horizontal pleiotropy tests of trigeminal neuralgia on forty-one inflammation cytokines.**

| **Outcome** | **Q1 pval** | **Q2 pval** | **intercept** | **Intercept pval** | **MR-PRESSO**  **Global** |
| --- | --- | --- | --- | --- | --- |
| B_NGF | 0.775 | 0.835 | 0.014 | 0.677 | 0.845 |
| CTACK | 0.056 | 0.086 | -0.008 | 0.854 | 0.108 |
| EOTAXIN | 0.910 | 0.844 | 0.025 | 0.271 | 0.851 |
| FGF_BASIC | 0.076 | 0.117 | 0.003 | 0.934 | 0.109 |
| G_CSF | 0.719 | 0.668 | 0.024 | 0.291 | 0.698 |
| GROA | 0.740 | 0.721 | -0.032 | 0.351 | 0.728 |
| HGF | 0.275 | 0.283 | 0.021 | 0.388 | 0.309 |
| IFN_G | 0.412 | 0.422 | 0.021 | 0.367 | 0.430 |
| IL_10 | 0.772 | 0.814 | 0.013 | 0.552 | 0.813 |
| IL_12_P70 | 0.605 | 0.696 | 0.007 | 0.749 | 0.702 |
| IL_13 | 0.661 | 0.543 | -0.044 | 0.205 | 0.568 |
| IL_16 | 0.349 | 0.368 | 0.030 | 0.400 | 0.368 |
| IL_17 | NA | 0.344 | NA | NA |  |
| IL_18 | 0.279 | 0.295 | 0.030 | 0.411 | 0.310 |
| IL_1B | 0.660 | 0.706 | 0.023 | 0.514 | 0.727 |
| IL_1RA | 0.898 | 0.870 | -0.031 | 0.350 | 0.890 |
| IL_2 | 0.565 | 0.630 | -0.019 | 0.560 | 0.666 |
| IL_2RA | 0.971 | 0.984 | 0.012 | 0.719 | 0.978 |
| IL_4 | 0.109 | 0.138 | 0.016 | 0.577 | 0.150 |
| IL_5 | 0.820 | 0.871 | -0.015 | 0.665 | 0.867 |
| IL_6 | 0.490 | 0.489 | 0.021 | 0.347 | 0.526 |
| IL_7 | 0.388 | 0.437 | -0.024 | 0.493 | 0.476 |
| IL_8 | 0.952 | 0.972 | -0.011 | 0.749 | 0.973 |
| IL_9 | 0.960 | 0.712 | -0.057 | 0.106 | 0.706 |
| IP_10 | 0.511 | 0.428 | -0.042 | 0.221 | 0.418 |
| M_CSF | 0.964 | 0.878 | -0.051 | 0.218 | 0.867 |
| MCP_1_MCAF | 0.352 | 0.449 | -0.005 | 0.821 | 0.431 |
| MCP_3 | 0.153 | 0.221 | 0.002 | 0.979 | 0.258 |
| MIF | 0.218 | 0.213 | -0.036 | 0.363 | 0.262 |
| MIG | 0.308 | 0.405 | 0.001 | 0.978 | 0.371 |
| MIP_1A | 0.523 | 0.440 | -0.042 | 0.223 | 0.434 |
| MIP_1B | 0.365 | 0.278 | 0.030 | 0.202 | 0.297 |
| PDGF_BB | 0.484 | 0.588 | -0.005 | 0.830 | 0.603 |
| RANTES | 0.255 | 0.215 | -0.043 | 0.274 | 0.222 |
| SCF | 0.876 | 0.927 | -0.003 | 0.887 | 0.941 |
| SCGF_B | 0.831 | 0.769 | -0.036 | 0.283 | 0.783 |
| SDF_1A | 0.817 | 0.615 | 0.035 | 0.149 | 0.632 |
| TNF_A | 0.710 | 0.793 | 0.008 | 0.798 | 0.789 |
| TNF_B | 0.591 | 0.720 | 0.021 | 0.808 | 0.727 |
| TRAIL | 0.952 | 0.972 | -0.007 | 0.731 | 0.962 |
| VEGF | 0.651 | 0.745 | 0.004 | 0.852 | 0.753 |

Q1 pval: p value of Q test from IVW method; Q2 pval: p value of Q test from MR-Egger method
